# Platelet Subpopulations in Health and Disease: Heterogeneity, Clinical Associations, and Therapeutic Targeting

**DOI:** 10.3390/cells15010011

**Published:** 2025-12-20

**Authors:** Deepa Gautam, Giovanni Goggi, Elisabeth M. Battinelli

**Affiliations:** 1 Division of Hematology, Brigham and Women’s Hospital, Boston, MA 02115, USA; fdeepa@bwh.harvard.edu; 2 Harvard Medical School, Boston, MA 02115, USA; 3 Department of Molecular Medicine, University of Pavia, 27100 Pavia, Italy; giovanni.goggi01@universitadipavia.it

**Keywords:** platelet subpopulations, reticulated platelets, aggregatory platelets, procoagulant platelets, coated platelets

## Abstract

Platelets are often described in simple terms as small anucleate cells that mediate hemostasis, but studies over more than half a century have shown that circulating platelets are heterogeneous in size, density, age, and functional responses. These subtypes not only contribute to normal hemostasis but also play pivotal roles in the pathogenesis of diverse diseases, including cardiovascular, inflammatory, and malignant disorders. Accumulating evidence indicates that alterations in specific platelet subtypes are closely linked to disease onset, progression, and severity, underscoring their importance as both biomarkers and potential therapeutic targets. Current clinical assessments of platelet status rely primarily on platelet count and mean platelet volume (MPV) as part of routine complete blood count analysis. However, these global measures often fail to capture differences in platelet subtypes, which may remain undetected despite their significant contribution to disease pathology. This gap highlights the necessity of moving beyond conventional metrics toward a more nuanced understanding of platelet heterogeneity and its clinical implications. In this review, we discuss the diversity of platelet subpopulations and their roles in health and disease, emphasizing how specific subsets contribute to divergent pathological mechanisms. We also highlight emerging strategies that target defined platelet subpopulations, illustrating how this knowledge could pave the way for more precise diagnostic and therapeutic approaches.

## 1. Background

Antiplatelet therapy is a cornerstone of treatment for many cardiovascular, thrombotic, and cerebrovascular disorders, yet in practice these drugs often fail to provide consistent protection [1,2,3,4]. A likely reason may be that current therapies are designed to broadly inhibit platelet activation while overlooking the underlying diversity of platelet subtypes, which differ in their activation profiles, receptor expression, and contribution to disease mechanisms. There is a need to integrate knowledge and awareness of platelet subtypes into clinical reasoning, as this would enable a precision medicine-based approach and support more tailored treatment strategies.

In this review, we first describe how platelet subpopulations were initially observed and how advances in technology have allowed their further characterization and categorization. We then outline the defining features of each subtype and their involvement in different disease conditions. Finally, we summarize current therapeutic approaches that specifically target platelet subtypes and discuss their potential for improving clinical outcomes.

## 2. Early Evidence of Platelet Heterogeneity

In the mid-20th century, emerging evidence revealed the structural, biochemical, and functional diversity of platelets, challenging the long-held view of them as a uniform population. In 1965, Webber and Firkin’s morphological study provided the first evidence of platelet heterogeneity by revealing two populations under osmotic stress [5]. When exposed to distilled water and examined under an electron microscope, one group appeared pale and damaged, with loss of organelles, while the other remained denser, retained its organelles, and exhibited pseudopodia. Serial sectioning confirmed that these were not transient states but different populations, possibly reflecting differences in platelet age or inherent biochemical properties [5].

Further studies in 1969 using density gradient centrifugation demonstrated that human platelets could be separated into large-heavy and light-small subtypes [6]. The large-heavy platelets had nearly twice the volume of the light-small ones and exhibited greater glycogen content, enhanced glycolysis and protein synthesis, and increased resistance to osmotic stress. This indicated that they were metabolically and functionally more active and might have contributed more substantially to overall platelet function. In contrast, smaller, lighter platelets showed reduced responsiveness, consuming more extracellular adenosine diphosphate (ADP) [7,8]. Around the same time, Ingram and Coopersmith described ribonucleic acid (RNA)-rich “reticulated” platelets (RPs) that increased after acute blood loss, paralleling reticulocytes in erythropoiesis [9]. Subsequent studies linked density with age and function. Using discontinuous Percoll gradients, denser platelets were identified as younger, confirmed by thiazole orange staining and in vivo biotinylation, whereas low-density platelets showed features of aging, including reduced RNA and diminished functional responses [10,11]. Together, these studies indicate that dense platelet fractions are enriched in younger, more reactive platelets, although density alone is not a perfect marker of age.

Platelet heterogeneity has also been linked to megakaryocyte ploidy and maturation. Mature megakaryocytes predominantly occur as 8n, 16n, and 32n and appear to generate platelets with different structures and densities. Platelets derived from 32n megakaryocytes are lighter and enriched in the surface-connected canalicular system, whereas those from 8n megakaryocytes are denser and larger with abundant granules and mitochondria [12]. Further support for a density-based aging model came from studies in rabbits, where platelet subpopulations were radiolabeled and tracked following separation by density using Stractan gradients. Dense platelets showed longer mean survival compared to less dense ones, suggesting that platelet density decreases with age in circulation. However, not all dense platelets transitioned to lighter forms over time, indicating that heterogeneity may also arise from differences at the level of platelet production [13]. Refinements in separation methods underscored additional complexity. A colloidal silica-polyvinylpyrrolidone system showed that platelet density populations are themselves heterogeneous in size. In both human and rat samples, dense platelets ranged from less than 3 femtoliters (fL) to more than 21 fL, while light platelets did not exceed 13 fL [14]. Cohort labeling with 75Se-selenomethionine showed no significant shift in buoyant density with aging. The higher specific activity in dense, younger platelets was attributed to protein synthesis inherited from their megakaryocyte precursors, supporting the view that dense, intermediate, and light platelets can originate simultaneously from megakaryocytes of differing ploidy [14].

Subcellular and functional analysis of density-separated platelets further demonstrated functional divergence among platelet subpopulations. Using isosmolar arabinogalactan gradients, platelets were isolated and assessed for organelle content. Mitochondria and lysosomes showed minimal variation across density fractions, while dense bodies and serotonin content declined significantly with decreasing density. Among alpha granule (⍺-granule) components, platelet factor 4 (PF4) showed a sharp density-dependent decrease, whereas beta-thromboglobulin was comparatively stable [15]. Under arterial-flow conditions, high-density platelets adhered to collagen up to fourfold faster than low-density platelets and were less sensitive to prostacyclin. Flow cytometry revealed that high-density platelets expressed higher levels of the collagen receptor GPI⍺/II⍺, which may explain their enhanced adhesion [16]. Two platelet populations distinguished by phosphotyrosine phosphatase (PTPase) activity were also identified; PTPase-negative platelets reacted more rapidly during hemostasis and predominated in early collagen adhesion at high flow [17].

Not all findings favored a density-based aging model. In studies tracking 51Cr-labeled human platelets, radioactivity distributions across heavy and light fractions remained stable over time, suggesting comparable survival among density classes and supporting Paulus’ view that heterogeneity is shaped more by thrombocytopoiesis than by in-circulation aging [18]. A theoretical analysis of platelet density separation highlighted the physical limitations and inconsistencies in commonly used centrifugation methods. Platelet density separation was limited by the physical constraints of centrifugation methods. Continuous gradients allowed a more accurate approach to equilibrium, whereas discontinuous gradients trapped subpopulations at interfaces and distorted density distribution. Inadequate centrifugation duration or gradient design therefore misclassified subpopulations, leading to size-based rather than density-based distinctions [13].

Megakaryocytes (MKs) themselves display considerable cellular heterogeneity across developmental stages and anatomical sites, and it is therefore plausible that this variability is transmitted to the platelets they generate [19]. RNA-sequencing studies on mouse and human MKs have found the existence of at least 3 functionally diverse subpopulations of megakaryocytes that possess gene signatures related to platelet generation, hematopoietic stem cell niche interaction, and inflammatory responses [20]. MKs differentially sort and package protein cargo into α-granule subpopulations that are preserved during proplatelet extension and platelet production, resulting in platelets with heterogeneous granule contents [21].

Aging induces sex-specific remodeling of MK subpopulations characterized by pro-thrombotic and pro-inflammatory signatures, and this parallels the heightened platelet activation and inflammation occurring with aging, increasing thrombotic and cardiovascular risk [22,23,24]

Taken together, prior work indicates that platelet diversity arises from both intrinsic differences established during production by megakaryocytes and dynamic changes acquired during circulation. These classical studies therefore provide the foundation for the concept of density- and age-based platelet subtypes, in which younger, denser, more reactive platelets can be distinguished from older, less dense, hyporeactive platelets. Building on these classical observations, recent methodical approaches have refined the mapping of platelet phenotypes and defined age- and activation-related subsets relevant to specific disease settings, as discussed in the following sections.

## 3. Progress in Understanding Platelet Subpopulations Through Recent Advances

Classical density-based and morphological studies provided only a coarse view of platelet heterogeneity. More recently, new high-dimensional and single-cell techniques have allowed a more detailed analysis of platelet characteristics. These methods measure several markers on the same platelet and have made it possible to map platelet phenotypes in greater detail, separate young from old platelets, and reveal activation-linked subpopulations such as aggregatory, procoagulant, and coated platelets.

Multicolor flow cytometry has deepened insight into platelet diversity. A six-color protocol revealed three subpopulations after strong stimulation with two agonists. These were normal-sized platelets, smaller platelets, and platelet fragments. The pattern remained stable even at maximal activation, which shows that platelet responses are not uniform. Normal-sized platelets showed features of aggregation, such as PAC1 binding and preserved mitochondrial function. The smaller subsets displayed a procoagulant profile with Annexin V (AV) binding and loss of mitochondrial potential [25].

Mass cytometry has enabled high-dimensional analysis of surface protein at the single-cell level. Application of a 14-antibody metal-tagged panel revealed previously unrecognized subpopulations, allowing comparative analysis between a Glanzmann thrombasthenia patient and healthy donors. The approach identified previously unrecognized platelet subpopulations in healthy donors, highlighting functional heterogeneity within the circulating pool. In Glanzmann thrombasthenia there was lower expression of CD41, CD61, and activated integrin αIIbβ3. It also showed broader shifts with higher CD9, CD42a, and CD63 and lower CD31, CD154, and GPVI, suggesting broader phenotypic changes beyond the primary integrin deficiency [26].

Multicolor flow cytometry combined with computational analysis has mapped platelet subpopulations and their regulation. Upon stimulation with a strong agonist in whole blood, three subtypes appeared that differed in phosphatidylserine (PS) exposure and fibrinogen binding. All groups showed enhanced CD62P expression and partial shedding of CD42b. Prostacyclin reduced procoagulant markers and produced four new subpopulations with varied activation features, linked to reduced mitochondrial depolarization, a key step in the procoagulant response [27].

Imaging flow cytometry has shown changes created by storage. During cryopreservation with DMSO, platelets developed specific phenotypes that included procoagulant and apoptotic profiles. A novel subset appeared that expressed PS and CD42b but lacked PAC1 and CD62P, suggesting a state not aligned with typical aggregatory or apoptotic platelets. This subset may help explain the stronger clotting seen with cryopreserved product. These findings emphasize that platelet subpopulations can be shaped not only by physiological activation but also by external factors such as storage [28].

A 16-parameter spectral flow cytometry panel paired with machine learning provided detailed single platelet phenotyping across many agonists. Changes in CD62P, PAC1, CD63, and CD42b marked different activation profiles. Classic clustering did not yield sharp boundaries, yet patterns suggested that some platelets are predisposed to degranulation or receptor shedding. Machine learning algorithms identified activation states and separated young from old platelets using size, granularity, and surface protein expression. Young platelets consistently showed higher expression of activation markers, reinforcing the link between platelet age and functional potential [29].

Single-cell transcriptomic studies with machine learning have mapped platelet diversity across inflammatory disease. A specific subtype of platelets was enriched in patients with fatal outcomes and characterized by transcriptional programs linked to coagulation, hypoxia, and oxidative stress. Shared transcriptional signatures appeared across conditions such as COVID-19, sepsis, and systemic lupus erythematosus, which points to common pathways of platelet-mediated immunopathology. Analyses of platelet contacts with monocytes and lymphocytes also showed a role in shaping immune activation beyond hemostasis [30,31].

Together, these technological advances provide the experimental background for the age-based and activation-linked platelet subtypes described in the following section.

## 4. Platelet Subpopulations Based on Age and Activation: Characteristics and Role in Diseases

Platelet heterogeneity has been classified using several parameters, including density, age, and functional reactivity. Based on these characteristics, platelet subpopulations are commonly described as RPs and as subsets defined by activation patterns, such as aggregatory, procoagulant, and coated platelets.

### 4.1. Reticulated Platelets

RPs are the youngest circulating platelets and mark active thrombopoiesis. Megakaryocytes extend proplatelets into the sinusoid, which form pre-platelets that can revert to proplatelets or elongate into barbell-shaped intermediates. Fission of these barbells yields two immature RNA-rich platelets that enter the blood [32,33].

Early work proposed that larger platelets are younger, but increased size mainly reflects higher megakaryocyte ploidy during accelerated marrow turnover. Under steady-state production, size and age diverge. In healthy adults, analysis with the Abbott CELL-DYN Sapphire multiparameter hematology analyzer by FACSCalibur (Becton Dickinson, Franklin Lakes, NJ, USA), which uses MAPSS optical scatter with fluorescence for complete blood counts and optical platelet counting, showed an inverse relationship between RP and MPV, indicating that larger platelets do not signify younger platelets under steady-state conditions [7,34,35].

Intracellular density emerges as a more reliable marker of platelet age and reactivity. With aging, platelets show ultrastructural deterioration, including loss of cytoskeleton, which can influence buoyant density [36]. Morphological and ultrastructural studies further support the unique features of RPs. Transmission electron microscopy showed that RPs more frequently exhibit pseudopodia and a non-discoid shape—features that are a platelet activation marker. RPs also present a more complex intracellular architecture with a fully developed Golgi apparatus, rough endoplasmic reticulum, and higher number of alpha-granules, dense granules, and mitochondria than older platelets [37].

**Figure 1:**
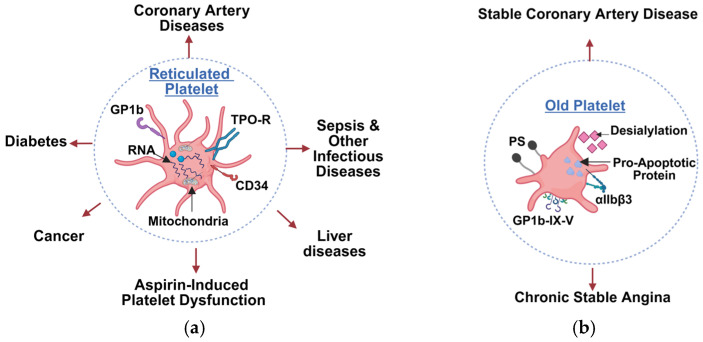
Classification of platelet subtypes based on age-related phenotypes and their associated disease contexts. (**a**) Young or reticulated platelets (RPs). (**b**) Old platelets. RPs, given their more complex cellular composition and architecture, are more involved in multiple diseases, including coronary artery disease, diabetes, sepsis, aspirin-induced platelet dysfunction, liver diseases and cancer. In contrast, older mature platelets, being less reactive, are less involved. GP1b, glycoprotein 1b; RPs, reticulated platelets; TPO-R, thrombopoietin receptor; RNA, ribonucleic acid; PS, phosphatidylserine; CD34, cluster of differentiation 34.

One of the key distinctions of RPs is their high RNA content (Figure 1a). Platelets inherit a finite RNA pool from megakaryocytes that degrades with age. Bulk RNA content decreases over time, while specific transcripts follow regulated patterns [38]. Younger platelets are enriched in transcripts linked to thrombin, thromboxane, and GP ⍺IIbβ3 signaling. They also retain higher levels of transcripts coding for key surface integrins involved in hemostasis, with increased P-selectin expression upon ADP or thrombin receptor activating peptide (TRAP) stimulation. RPs also have more transcripts related to calcium homeostasis, such as stromal interaction molecule 1 (STIM1), Orai1, and Orai2, which are crucial for platelet activation pathways [39]. With aging, ribosomal signatures such as 18S and 28S rRNA decline, and total protein content drops to about one half, including lower levels of integrin subunit alpha 2b (ITGA2B), integrin subunit beta 3 (ITGB3), STIM1, and P selectin [38,40]. RPs that display higher levels of human leukocyte antigen/major histocompatibility complex (HLA I/MHC-I complex) have higher glycoprotein VI (GPVI) levels than old platelets [41].

Several studies have examined whether transcript-level differences in platelet subpopulations are reflected at the protein level. In healthy donors, platelets sorted by thiazole orange fluorescence as a surrogate of mRNA content showed striking age-related differences in the proteome. Old, thiazole orange dim platelets contained about 45% less total protein than young platelets and were depleted in cytoskeletal and mitochondrial proteins, with pathway analyses indicating enrichment of apoptosis and senescence, while young platelets were enriched for hemostasis-related pathways. Functionally, old platelets displayed impaired spreading and adhesion, reduced calcium mobilization, and diminished granule secretion, directly linking age-dependent RNA content to altered proteome and function [38]. Complementary work using washed platelets and data independent acquisition mass spectrometry in young, middle-aged and elderly individuals showed that the platelet proteome remains relatively stable from young to middle age but diverges in the elderly. Platelets from older subjects exhibited clusters of differentially expressed proteins related to complement activation, phagosome pathways, hemostasis, and platelet degranulation, with additional enrichment of calcium and NF kappa B signaling, providing a proteomic basis for platelet hyperreactivity and increased thrombotic susceptibility in aging populations [42].

All these features suggest that RPs, in general, present a more reactive phenotype compared to the older platelets. This can be due to the greater abundance of constitutional surface receptors that allow RPs to be more sensitive upon agonist stimulation. Platelets of <24 h old in circulation respond to stimuli with greater calcium flux and degranulation and contribute more to the formation of thrombi in vitro and in vivo. The age-associated decrease in thrombotic function was accompanied by significant decreases in the surface expression of GPVI and CD31 [PECAM-1] [43].

In clinical practice RPs are often reported either as the Immature Platelet Count [IPC#], expressed as an absolute number, or as the Immature Platelet Fraction [IPF %], reported as a percentage of the total platelet count. These measures, which overlap with the thiazole orange bright population, are available on automated analyzers, most commonly Sysmex. The most widely used platforms are Sysmex XE-2100 and XN series analyzers. Both use a fluorescent platelet channel in which an RNA-sensitive dye stains platelets, and the instrument measures size and fluorescence to distinguish immature from mature platelets to derive IPF and IPC. Earlier XE-series analyzers obtain IPF from a reticulocyte channel that uses a polymethine dye, whereas XN analyzers use a dedicated platelet fluorescence (PLT-F) channel with an oxazine dye and a longer counting time, which gives slightly higher reference intervals and improved precision at low platelet counts [44]. As a result, IPF reference intervals depend not only on age, sex, ethnicity, and geography but also on analyzer type. For example, the XE-2100 reports approximately 0.5–3.3% in healthy adults, while XN series analyzers report about 1.0–7.3%, with similar ranges described in other cohorts [45].

#### Reticulated Platelets in Health and Disease

RPs are elevated across many patient groups, especially in cardiovascular disease. RPs recruited to thrombi under high shear have higher RNA content, increased integrin ⍺IIbβ3 expression, and generate more prothrombinase, which makes them prone to early thrombus formation [46]. In coronary artery disease, immature platelet measures help with risk assessment. RPs rise stepwise from unstable angina to non-ST-segment elevation myocardial infarction (NSTEMI) to ST-segment elevation myocardial infarction [STEMI], and the immature platelet fraction is highest in acute events, especially in STEMI [47]. Even in stable CAD, increased IPF, MPV, and platelet distribution width (PDW) have been reported, suggesting enhanced platelet turnover [48]. Abnormal increases in platelet size have also been associated with myocardial infarction [49]. Experimental models further showed that accelerated atherogenesis is preceded by increased megakaryocyte size and ploidy, producing larger, more reactive platelets [50].

RPs have been shown to be actively involved in diabetes pathogenesis, a phenomenon indicated by elevated MPV [51,52]. Hyperglycemia triggers neutrophil release of S100A8/A9, which activates Kupffer cells and induces IL-6-dependent thrombopoietin (TPO) production in the liver. As a result, megakaryopoiesis accelerates, boosting RP production. This, in turn, raises thrombotic risk and diminishes the effectiveness of standard antiplatelet therapy [52]. In type 2 diabetes patients, platelet and RP counts are higher, with increased monocyte-platelet aggregates. RP levels correlated with hemoglobin A1c (HbA1c) and higher plasma S100A8/A9 [53].

High turnover and a large pool of young platelets also limit the effect of antiplatelet drugs. Patients with many RPs and larger MPV show less inhibition by aspirin and by dual antiplatelet therapy, and high on-treatment reactivity predicts events such as stent thrombosis [54,55,56]. A second mechanism involves cyclooxygenase-2 (COX-2) expression in RPs, which sustains thromboxane A_2_ generation (TxA_2_) despite aspirin therapy. COX-2 is upregulated in high turnover states such as immune thrombocytopenia, multiple myeloma, or post-cardiac surgery [57,58,59]. Similar patterns appear in essential thrombocythemia and coronary disease [60]. Increased platelet turnover may induce COX-2 expression in megakaryocytes, driven by GATA-1-dependent regulation of hematopoiesis and inflammatory cytokines released after cardiac surgery, leading to COX-2-positive platelets [58].

Beyond thrombosis, RPs have been reported to play an important role in cancer. Emerging evidence indicates that RPs carry programmed cell death ligand 1 (PD-L1) and transfer PD-L1 to circulating leukocytes through platelet-leukocyte adhesion, inflating apparent PD-L1 levels on monocytes and lymphocytes [61]. This RP-mediated PD-L1 loading provides a circulating source of checkpoint signaling that dampens T-cell activity. In patients with active thrombopoiesis, a higher burden of PD-L1-positive RPs is associated with improved responsiveness to anti-PD-1 therapy. Accordingly, quantifying PD-L1 on RPs offers a practical blood biomarker and, when combined with tumor PD-L1 and tumor proportion score (TPS), improved prediction of response to anti-PD-1 therapies [61]. Building on this concept of platelet-mediated immune modulation, a previous study shows that platelets can directly interact with cancer cells and internalize their DNA. Uptake occurs both as free DNA strands and via extracellular vesicles (EVs), relying on non-clathrin and clathrin-mediated pathways, respectively. Notably, platelet activation is required for DNA release but not for DNA acquisition. These observations underscore a dynamic bidirectional exchange between platelets and their microenvironment and raise the question of whether phagocytic or endocytic capacity differs among platelet subpopulations—particularly reticulated platelets. Defining subset-specific uptake mechanisms could provide new insights into functional platelet heterogeneity and clarify how distinct platelet populations contribute to immune regulation, cancer progression, and thrombosis [62]. In cirrhosis, RPs are increased and more active. In a prospective cohort of 45 patients without hepatocellular carcinoma, flow cytometry and immunofluorescence showed higher RPs percentages than in healthy controls. Higher baseline RPs predicted progression to further decompensation or acute-on-chronic liver failure [63]. In thrombocytopenic patients with liver dysfunction, immature platelet fraction (IPF%) measured on routine analyzers, combined with platelet count, distinguished cirrhosis from other chronic liver diseases better than either metric alone [64]. In prostate cancer, elevated levels of RPs have been associated with micrometastatic disease and may support circulating tumor cell (CTC) survival, extravasation, and early metastatic spread. Their mobilization from the bone marrow appears to be tumor-driven, enhancing tumor cell survival and invasiveness through direct interactions and the release of pro-survival factors. Importantly, high levels of this platelet subpopulation independently predict early biochemical recurrence within the first year after prostatectomy, highlighting their potential as a prognostic biomarker and therapeutic target [65].

COVID-19 patients have been shown to have higher immature platelet indices than stable cardiovascular controls. On admission, IPF% and immature platelet count in COVID-19 were like acute myocardial infarction and higher than stable controls. Over the disease course, the peak IPF% matched acute myocardial infarction, while the peak IPC was higher than both comparison groups. These findings point to an expansion of immature RPs that may contribute to the high venous and arterial thrombotic risk in COVID-19 [66].

In suspected infection, RPs percentage was elevated and showed the highest diagnostic accuracy [67]. In critically ill adults who were not septic at intensive care unit (ICU) entry, the IPF% identified those who later developed sepsis. IPF% rose before clinical diagnosis, tracked inversely with platelet count, and fell after onset. Since IPF% is available on routine analyzers, it offers a practical early marker to monitor in the ICU [67,68]. IPF correlated positively with human immunodeficiency virus (HIV) viral load, reflecting compensatory megakaryocyte activity in HIV-related thrombocytopenia [69].

### 4.2. Functional Subtypes of Activated Platelets and Their Roles in Disease

Upon activation, platelets do not respond in a uniform manner but instead differentiate into different functional subtypes, each contributing uniquely to thrombus formation and vascular response. Experimental work in intravital and flow-based models shows that platelets engaged predominantly in aggregation form densely packed fibrin(ogen)-binding aggregates, whereas separate patches of platelets expose phosphatidylserine, inactivate αIIbβ3, and bind coagulation factors, thereby acting in a mainly procoagulant manner [70,71]. Depending on the signaling pathways engaged, activated platelets may assume an aggregatory, procoagulant, or coated phenotype, each defined by specific surface markers, intracellular events, and biological roles [72]. The following sections describe the mechanisms that drive the formation of these subpopulations and highlight their relevance in both physiological hemostasis and pathological conditions (Table 1).

#### 4.2.1. Aggregatory Platelets

**Figure 2:**
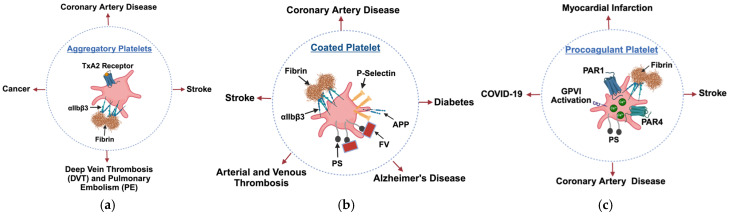
Classification of platelet subtypes based on functional phenotypes and their associated disease contexts. (**a**) Aggregatory platelets. (**b**) Coated platelets. (**c**) Procoagulant platelets. TxA_2_, thromboxane A_2_; PS, phosphatidylserine; FV, Factor V; APP, amyloid precursor protein; PAR1, protease-activated receptor 1; PAR4, protease-activated receptor 4; GPVI, glycoprotein VI.

Aggregatory platelets represent a functionally divergent subpopulation that contributes to the mechanical assembly of thrombi. Upon activation, these platelets express the high-affinity form of the integrin αIIbβ3, which binds to fibrinogen and supports platelet-platelet adhesion (Figure 2a). This interaction is the primary mechanism driving platelet cohesion under venous and moderate arterial shear conditions. It leads to the formation of stable platelet aggregates that form the core of the thrombus [73,74].

Studies using live imaging under flow have demonstrated that aggregatory platelets form tightly packed clusters with sustained integrin activation. These platelets retain membrane integrity and do not expose PS on their surface. Their role is primarily structural rather than enzymatic. Under higher shear conditions, particularly in narrowed arteries, the contribution of von Willebrand factor (vWF) becomes more prominent. vWF works together with fibrinogen to support the continued growth of the thrombus [74]. Beyond the classical role of integrin αIIbβ3 in mediating fibrinogen-dependent platelet aggregation, earlier studies have identified P-selectin as a key stabilizer of platelet aggregates [75]. Its surface expression has been shown to correlate with aggregate size, and functional inhibition of P-selectin significantly reduces both the number and size of platelet aggregates. Interestingly, this disaggregating effect occurs even when P-selectin is blocked several minutes after initial activation, indicating that its role is not in initiating aggregation but in stabilizing platelet-platelet contacts after fibrinogen binding [75].

Evidence from intravital and in vitro imaging studies has demonstrated a functional and spatial separation between aggregatory and non-aggregatory platelet populations during thrombus formation. Using high-resolution two-photon fluorescence microscopy, fibrinogen-binding platelets were shown to form compact aggregates, while PS-exposing platelets localized to distinct, nonaggregate regions of the thrombus [71]. Extending these mechanistic insights, high-dimensional single-cell profiling using mass cytometry has characterized aggregatory platelets as a subpopulation with sustained integrin activation, active α-granule secretion, and preserved membrane integrity. This phenotype not only supports their adhesive function but also promotes interactions with monocytes, indicating a possible role in linking thrombus formation with inflammatory processes [76]. Further delineating platelet heterogeneity, single-cell transcriptomic profiling has identified subpopulations consistent with an aggregatory phenotype. One such cluster exhibited elevated expression of genes involved in integrin signaling, cytoskeletal dynamics, and granule secretion, supporting a specialized adhesive role. Notably, this subset showed differential sensitivity to antiplatelet therapy, with reduced responsiveness to aspirin but enhanced inhibition via P_2_Y_12_ blockade [27]. Consistent with these observations, intravital microscopy of arteriolar injury in mice has shown that thrombi can organize into a densely packed core of P-selectin positive platelets that support thrombin generation and fibrin formation, surrounded by a shell of less activated, P-selectin negative platelets that are maintained predominantly by ADP-dependent signaling. Complementary high-resolution imaging and ultrastructural studies of jugular vein puncture wounds have described vaulted thrombi in which degranulated platelets line the interior of the thrombus and are covered by a layer of less activated platelets, further supporting a core and shell organization of functionally separate platelet subpopulations [70].

Altogether, these findings reinforce the role of aggregatory platelets as central structural components of the thrombus, with diverse molecular features that may influence thrombus growth and stability in both physiological and pathological settings.

##### Aggregatory Platelets in Health and Disease

In patients with acute myocardial infarction, immune complexes isolated shortly after symptom onset showed a strong capacity to promote platelet aggregation. Composed of C1q-binding antigen-IgG aggregates purified from serum, these complexes activate platelets predominantly through FcγRIIa with additional input from platelet-associated complement (C1q, the classical-pathway initiator, and C3, the central complement component). They also modified the responsiveness of healthy donor platelets to the inhibitory actions of prostacyclin and prostaglandin D_2_, shifting them toward a hyperreactive state. In contrast, immune complexes from patients with pulmonary cancer produced no significant effect, indicating a disease-specific mechanism. The antigenic component of these immune complexes is likely key to initiating platelet activation, highlighting an important interplay between immune responses and the development of an aggregatory platelet phenotype in cardiovascular disease [77].

Aggregatory platelets not only recruit additional platelets to sites of vascular injury but also interact with leukocytes, influencing hemostasis and inflammation. On potent thrombogenic surfaces such as fibrillar type I collagen, primary adherent platelets initially provide a highly reactive surface for platelet recruitment. Over time, calcium-dependent signaling reduces platelet adhesiveness by down-regulating integrin αIIbβ3, while procoagulant activity increases, marked by surface exposure of phosphatidylserine, reflecting the transition from a proaggregatory to a procoagulant phenotype. These procoagulant platelets, despite losing the ability to aggregate, enhance neutrophil adhesion and spreading, partly mediated by platelet-activating factor (PAF). Platelet-neutrophil interactions are increasingly recognized in the pathogenesis of ischemia/reperfusion injury, atherosclerosis, respiratory distress syndrome, and septic shock, making PAF modulation a potential therapeutic target [78].

#### 4.2.2. Coated Platelets

The discovery of collagen- and thrombin-activated (COAT) platelets marked a significant advancement in understanding platelet subpopulations and their role in coagulation. A previous study showed that Fc receptor gamma chain (FcRγ), an adaptor protein associated with the collagen receptor GPVI, is essential for the generation of coated platelets. Mice lacking FcRγ showed markedly reduced formation of coated platelets, diminished AV binding, and impaired platelet procoagulant function following dual agonist stimulation. In contrast, the absence of transglutaminase factor XIII A had no impact on coated platelet formation [79]. Coated platelets are known to develop through a sequence of events involving early activation of integrin ⍺IIbβ3, thrombin signaling, and the action of transglutaminase enzymes such as factor XIII [80].

COAT platelets, accounting for approximately 30% of the activated platelet population, retain α-granule proteins on their surface while exposing negatively charged phospholipids, creating an environment highly favorable for thrombin generation. Functionally, these platelets preferentially bind factor Xa (FXa), sustain platelet-derived Factor V (FV) activity, and enhance prothrombinase activity, making them crucial for stable clot formation (Figure 2b). This subset also plays a regulatory role in coagulation by serving as a surface for the expression of tissue factor pathway inhibitor (TFPI), a key physiological inhibitor of tissue factor (TF) [81]. Additionally, younger platelets appear more likely to become COAT platelets, suggesting an age-dependent regulation of their formation. This study fundamentally changed the understanding of platelet heterogeneity, highlighting that platelets are not uniform in function but instead exist in specialized subpopulations with different hemostatic roles [82]. Subsequent research expanded on this concept by uncovering the role of serotonin in COAT platelet formation. It was found that tissue transglutaminase and factor XIIIa (FXIIIa) mediate the covalent linking of serotonin to procoagulant proteins, either derived from platelet granules or plasma, which then bind to fibrinogen or thrombospondins (TSPs) on the platelet surface. This serotonylation process enhances the procoagulant properties of COAT platelets, linking platelet activation to a broader regulatory mechanism. Moreover, serotonin within the platelet cytoplasm was shown to influence small GTPases, which are critical regulators of granule secretion and platelet function. The physiological relevance of serotonin in platelet activation was further supported by studies in tryptophan hydroxylase KO (Tph-/-) mice, which have a significant reduction in peripheral serotonin. These mice exhibited prolonged bleeding times, suggesting that serotonin plays a key role in platelet function, particularly in the release of von Willebrand factor [VWF]. This finding reinforced the idea that COAT platelets are not only essential for hemostasis but may also contribute to pathological clotting conditions [83]. More recently, an in vivo venous thrombosis model demonstrated that the extent of thrombus formation depends on the serotonin transporter (SERT) rather than on platelet serotonin content: mice lacking SERT formed markedly fewer and smaller thrombi, whereas absence of platelet serotonin (Tph1-/- or SSRI-treated) did not reduce thrombus burden; neutrophil recruitment was also attenuated in SERT-deficient mice. These data suggest context-dependent roles for serotonin pathways in vivo and highlight transporter-dependent mechanisms in thromboinflammation [84]. Further, it was demonstrated that COAT platelets not only retain FV but also fibrinogen, VWF, TSPs, fibronectin, and α2-antiplasmin. The retention of these proteins is stabilized by serotonin, which binds covalently through transglutaminase-mediated cross-linking. Fibrinogen binding to these platelets appears to occur independently of classical GP ⍺IIbβ3 activation, as evidenced by the inability of PAC-1 and various GP ⍺IIbβ3 inhibitors to block its retention [85]. Several GP ⍺IIbβ3 antagonists, including eptifibatide, tirofiban, and DMP802, significantly increased the formation of coated platelets. A similar increase was observed in FcRT platelets, a related subpopulation generated through thrombin and Fc receptor activation [85]. Interestingly, serotonin was found to be covalently linked to fibrinogen, confirming its role in stabilizing platelet-bound clotting factors. Transglutaminase-mediated serotonylation covalently tethers α-granule proteins to the platelet surface and to fibrin/fibrinogen, creating multivalent, high-avidity attachments that keep procoagulant proteins in place, even in the presence of PAC-1 or potent αIIbβ3 antagonists. These findings suggest that COAT platelets only form under extreme hemostatic conditions, such as in response to severe vascular injury, where both collagen exposure and thrombin generation are sustained [86].

##### Coated Platelets in Health and Disease

Coated platelets have gained attention as important contributors to various disease processes including thrombotic, inflammatory, and neurodegenerative disorders.

Coated-platelet production differed markedly between stroke subtypes and healthy controls. A total of 60 stroke patients [20 lacunar, 40 cortical] and 70 controls were analyzed, revealing that lacunar stroke patients exhibited significantly lower coated-platelet levels compared to cortical stroke patients and controls. These findings indicate pathophysiological differences between stroke subtypes. These results support the lacunar hypothesis, indicating that lacunar strokes are primarily driven by small vessel disease rather than platelet-mediated thrombosis, as seen in cortical strokes [87]. In a subsequent study the relationship between coated-platelet levels and stroke recurrence was investigated in patients with nonlacunar ischemic stroke. Given that coated platelets support thrombin generation and are elevated in nonlacunar strokes, the study analyzed 190 patients, grouping them into tertiles based on coated-platelet levels, and monitored stroke recurrence over 12 months. The results demonstrated a significantly higher recurrence risk in patients with elevated coated-platelet levels, with recurrence rates of 17–18% in the middle and high tertiles compared to just 2% in the lowest tertile [88].

Elevated levels of coated platelets have been implicated not only in recurrent ischemic events but also in post-stroke cognitive decline. In a prospective study of patients with non-lacunar ischemic stroke, higher coated-platelet levels measured during the acute phase were significantly associated with lower Mini-Mental State Examination (MMSE) scores at three months post-infarction, independent of traditional vascular risk factors and stroke characteristics [89]. Another study assessed the predictive value of coated-platelet levels, for recurrent stroke and transient ischemic attack (TIA) in patients with lacunar stroke. A total of 109 patients with acute lacunar stroke were enrolled and monitored for up to 12 months. The study found that patients with elevated coated-platelet levels [≥42.6%] had increased hazard ratio for recurrent stroke or TIA compared to those with lower levels. Additionally, smoking was identified as a key factor associated with higher coated-platelet levels [90]. Conversely, another study demonstrated that patients with non-lacunar ischemic stroke who had lower levels of coated platelets were at increased risk of major hemorrhagic complications after discharge [91]. A study involving 271 stroke patients found that individuals with coated platelet levels below 36.5 percent had a significantly increased incidence of intracerebral and major extracranial bleeding within 12 months. In contrast, those with levels above this threshold experienced a markedly lower bleeding risk [91]. Evidence suggests a strong association between reduced coated platelet levels and the severity of spontaneous intracerebral hemorrhage. Patients with this condition had significantly lower coated platelet levels compared to healthy individuals [92]. In a separate cohort of 45 patients, coated platelet levels were inversely correlated with hemorrhage volume, indicating that lower levels were associated with larger bleeds. Together, these findings suggest that impaired coated platelet production may contribute to the extent of intracerebral bleeding and could serve as a marker for hemorrhagic severity [93].

Clinical studies have shown that coated platelet levels are persistently elevated in individuals with a history of mild traumatic brain injury (mTBI). In a cohort of veterans, coated platelet levels remained significantly higher than in matched controls, with no decline observed even up to nine years post-injury. These levels were not influenced by injury frequency, time since trauma, or comorbidities such as post-traumatic stress disorder or headache. Interestingly, coated platelet levels in mTBI patients were higher than those seen in acute ischemic stroke, suggesting a prolonged prothrombotic state [94].

Coated platelets have been identified as a promising peripheral biomarker for predicting the progression from mild cognitive impairment to Alzheimer’s disease. In a prospective study involving 74 patients with amnestic mild cognitive impairment, individuals with higher baseline levels of coated platelets showed a significantly increased risk of progressing to Alzheimer’s disease (AD) over a median follow-up of 24 months. Specifically, 37% of patients in the highest tertile of coated platelet levels progressed to AD, compared to only 4% in the lowest tertile. The hazard rate for progression was over five times higher in the upper tertile group. Although the underlying mechanism remains unclear, elevated coated platelet levels may reflect early amyloid-related or inflammatory changes associated with ADs pathology [95,96].

A significant subset of patients with bleeding disorders of unknown cause (BDUC) show a reduced capacity to generate coated platelets, despite normal results on standard platelet function tests [97]. Flow cytometry analysis revealed that approximately 23% of BDUC patients across three cohorts had low levels of coated platelets. These platelets play a critical role in thrombin generation and are characterized by the surface retention of ⍺-granule proteins through a serotonin- and transglutaminase-dependent mechanism. The study also found that desmopressin (DDAVP) can selectively enhance the production of coated platelets in these patients. This response was independent of VWF or FVIII levels [97]. A flow cytometry-based study evaluated the diagnostic value of coated platelet measurement in patients with unexplained bleeding symptoms. In a group of 101 patients with elevated bleeding scores, approximately thirty percent showed coated platelet levels below the reference range, while only two percent of healthy controls had similarly low levels. A clear association was observed between lower coated platelet levels and greater bleeding severity, even though routine platelet function tests appeared normal [98].

#### 4.2.3. Procoagulant Platelets

Differing from the coated platelet population described above, another subset of highly activated platelets is termed procoagulant platelets. Although coated and procoagulant platelets share key properties, most notably surface exposure of PS and the ability to support tenase and prothrombinase assembly and thrombin generation, procoagulant platelets expose PS and support thrombin generation, without requiring the stable serotonin and transglutaminase dependent surface coat of retained alpha granule proteins that characterizes coated platelets [86,99,100,101]. Procoagulant platelets represent a subpopulation of activated platelets that arise following strong stimulation, typically through simultaneous engagement of thrombin and collagen receptors [102]. Unlike traditional activated platelets, which support aggregation and granule release, procoagulant platelets are defined by a unique set of features, including sustained elevation of cytoplasmic calcium [103], loss of mitochondrial membrane potential with surface exposure of PS [104], inactivation of fibrinogen receptors [105], and the retention of procoagulant proteins such as fibrinogen and FV on their surface [82,86]. Notably, only a fraction of platelets undergoes this transformation under dual agonist stimulation, suggesting a bifurcation in the platelet response that remains incompletely understood [25]. These platelets serve as an efficient platform for the assembly of coagulation factor complexes, highlighting their critical role in thrombin generation and clot stabilization.

Procoagulant platelets were initially believed to form through two different mechanisms, one involving classical platelet activation and the other driven by apoptotic signaling. Apoptosis-associated procoagulant platelets exhibit features such as PS exposure, membrane blebbing, and microvesiculation, closely resembling apoptotic cells. Activation of the intrinsic apoptotic pathway using BCL2 homology 3 (BH3) mimetics like ABT-737 promotes thrombin generation and PS exposure in a manner dependent on the pro-apoptotic proteins Bak and Bax, as well as caspase activity. This pathway operates independently of traditional platelet activation mechanisms and does not require extracellular calcium [82,106]. In contrast, the conventional agonist-driven pathway relies on calcium influx and receptor-mediated activation but remains intact in the absence of Bak/Bax or caspase function. These findings highlight the dual regulatory routes leading to the procoagulant platelet phenotype and suggest that both apoptotic and non-apoptotic mechanisms contribute to platelet procoagulant activity under different physiological and pathological conditions [107]. While earlier studies emphasized apoptosis as a key mechanism more recent findings have identified a population of necrotic procoagulant platelets formed through a regulated cyclophilin D dependent process. These necrotic platelets rapidly expose PS accumulate in thrombi and contribute significantly to fibrin formation even under aspirin therapy [108]. Together these observations establish that both apoptotic and necrotic pathways can give rise to procoagulant platelets each regulated by different molecular cues and possibly operating under specific physiological or pathological conditions.

Initial studies described PS exposure as a characteristic limited to a single subpopulation of platelets following strong activation. Instead, two diverse PS-expressing subpopulations were identified, differing in calcium dynamics, mitochondrial integrity, and functional capacity [107]. One subset is characterized by sustained high intracellular calcium, mitochondrial depolarization, loss of membrane integrity, and minimal aggregation-features typical of highly procoagulant platelets. The second subset maintains low intracellular calcium, preserves mitochondrial and membrane function, and exhibits both fibrinogen retention and active integrin αIIbβ3, supporting aggregation. The formation of this low-calcium PS-positive subset is regulated by integrin signaling and is impaired in platelets with defective αIIbβ3 function [107]. Sustained cytoplasmic calcium levels are necessary, but not sufficient, for high-level PS exposure in response to agonists. Increased mitochondrial calcium levels are a key signal initiating mitochondrial permeability transition pore (mPTP) formation and PS exposure (Figure 2c). Blockade of mitochondrial calcium entry specifically inhibits procoagulant activity without affecting aggregation or secretion, reinforcing the idea that intracellular pathways regulate the formation of PS-positive platelet subtypes [109]. The transition of activated platelets into a procoagulant state involves not only PS exposure but also the inactivation of integrin αIIbβ3, which prevents further aggregation. This inactivation is driven by sustained intracellular calcium elevation, leading to cleavage of key integrin-associated proteins such as the β3 subunit, talin, and Src kinase. Additional regulation involves transmembrane protein 16 F (TMEM16F)-mediated phospholipid scrambling and mPTP formation. Loss of TMEM16F or mPTP function reduces both PS exposure and integrin inactivation, highlighting a coordinated mechanism involving calcium signaling, membrane remodeling, and mitochondrial pathways in the development of the procoagulant phenotype [105].

##### Procoagulant Platelets in Health and Disease

Platelets play a central role in hemostasis, but their dysregulation contributes to the development and progression of cardiovascular diseases such as coronary artery disease (CAD). Increased levels of procoagulant platelets have been identified in patients with CAD using GSAO and P-selectin assays in whole blood. These platelets respond robustly to agonists such as thrombin and ADP, and their activity is not fully suppressed by standard antiplatelet therapies, including aspirin or clopidogrel. Although dual antiplatelet therapy (DAPT) provides partial modulation, it does not completely inhibit platelet responses to strong stimuli. These findings underscore the potential of procoagulant platelets as both biomarkers of thrombotic risk and promising therapeutic targets in CAD [110].

The role of procoagulant platelets was investigated in the diagnosis and understanding of heparin-induced thrombocytopenia (HIT), a prothrombotic immune disorder triggered by antibodies targeting platelet factor 4 (PF4)-heparin complexes. HIT plasma induced platelet procoagulant activity via FcγRIIa, a receptor activated by these antibodies, in a heparin-dependent process. The assay achieved a remarkable 98% diagnostic accuracy, positioning itself as a rapid and effective alternative to the traditional serotonin release assay (SRA). Furthermore, the study underscored the role of thrombin generation and FcγRIIa activation in perpetuating thrombotic risk in HIT, offering valuable insights for potential therapeutic interventions targeting procoagulant platelets [111].

PS-positive platelets and microparticles (MPs) played a role in the hypercoagulable state of colon cancer patients. By analyzing blood samples from 112 colon cancer patients [Stages I-IV] and 33 healthy controls, it was found that PS-exposing platelets increased progressively with cancer stage, while PS-positive MPs were significantly elevated in advanced stages [III/IV]. These PS+ platelets and MPs contributed to enhanced coagulation activity, as demonstrated by increased FXa and thrombin generation, shortened clotting time, and greater fibrin formation [112].

Procoagulant platelets have been shown to play a pivotal role in the development of venous thrombosis, including deep vein thrombosis (DVT) and pulmonary embolism (PE). Evidence from both human patient samples and murine DVT models demonstrates that these platelets are enriched within thrombi and circulate at elevated levels in patients with confirmed venous thromboembolism. Moreover, genetic deletion of key regulators of procoagulant activation, such as TMEM16F and cyclophilin D, significantly reduced thrombus formation in mice without compromising normal hemostasis [113].

Procoagulant platelets have also been recognized as mediators of trauma-induced platelet dysfunction [114]. In patients with severe injury, circulating platelets rapidly transitioned into ballooned procoagulant forms within minutes, releasing activated MPs that coated leukocytes. This transformation was driven by histone H4, a damage-associated molecular pattern abundantly released after tissue injury. When healthy platelets were exposed to histone H4 in vitro, they exhibited membrane ballooning and PS exposure, closely resembling the phenotype seen in trauma patients [114].

A procoagulant state is well established in diabetes mellitus and is thought to arise from activation of the intrinsic coagulation pathway, reduced fibrinolytic activity, and altered platelet function [115]. Compared to non-diabetic individuals, platelets from patients with diabetes display increased exposure of PS, greater binding of coagulation FVa, and enhanced production of platelet-derived MPs following stimulation [116]. These changes lead to a significantly higher platelet procoagulant index, accelerated thrombin generation, and shortened plasma clotting time. In type 2 diabetes, a condition known for its elevated thrombotic risk, circulating platelets exhibit a procoagulant phenotype even in the absence of external agonist stimulation. Studies using density gradient separation have identified a population of small platelets concentrated in medium dense fractions that display classical procoagulant traits. These include decreased expression of activated integrin ⍺IIbβ3, increased surface PS, higher levels of lysosome-associated membrane protein-1 (LAMP-1) indicating lysosomal degranulation and disrupted mitochondrial membrane potential. These characteristics closely resemble those of platelets exposed to strong in vitro agonist stimulation, suggesting that in type 2 diabetes a subset of platelets may undergo chronic or spontaneous activation leading to a sustained procoagulant state [117].

During the COVID-19 pandemic, procoagulant platelets emerged as critical mediators of the thromboinflammatory complications observed in severe SARS-CoV-2 infection. Platelets from COVID-19 patients exhibited a reduced ability to become procoagulant in response to strong agonists, as shown by decreased mitochondrial depolarization and PS exposure. Interestingly, mice lacking cyclophilin D, a key regulator of procoagulant platelet formation, showed accelerated mortality in a model of pulmonary microvascular thrombosis, indicating that impaired but dysregulated procoagulant responses may worsen thrombotic outcomes in vivo [118]. In contrast, procoagulant platelets have been implicated in the pathogenesis of vaccine-induced immune thrombotic thrombocytopenia, a rare but severe complication following ChAdOx1 nCoV-19 vaccination. Patients with this condition produced high-titer antibodies against PF4, which triggered platelet activation and induced the formation of procoagulant platelets through a PF4 and heparin-dependent mechanism. These platelets exhibited increased PS exposure and P-selectin expression, contributing to widespread arterial and venous thromboses [119]. Mechanistic studies have also shown that the phosphatidylinositol 3-kinase (PI3K)-protein kinase B (AKT) signaling pathway played a central role in the generation of procoagulant platelets in severe COVID-19. Platelets from critically ill patients displayed increased AKT phosphorylation, which correlated with surface PS and CD62p expression. This effect is mediated through FcγRIIA receptor signaling and occurs independently of GP ⍺IIbβ3 engagement [120].

## 5. Therapy Used to Target Platelet Subpopulations

The growing understanding of platelet heterogeneity highlights the need for a more tailored therapeutic approach, directed at the specific platelet subtype driving the pathological process, offering the possibility to maximize efficacy while limiting adverse effects. This precision strategy requires mapping the functional defects or hyperactivities of platelet subpopulations and linking them to selective pharmacological targets, thereby moving toward a subtype-oriented therapy.

**Figure 3:**
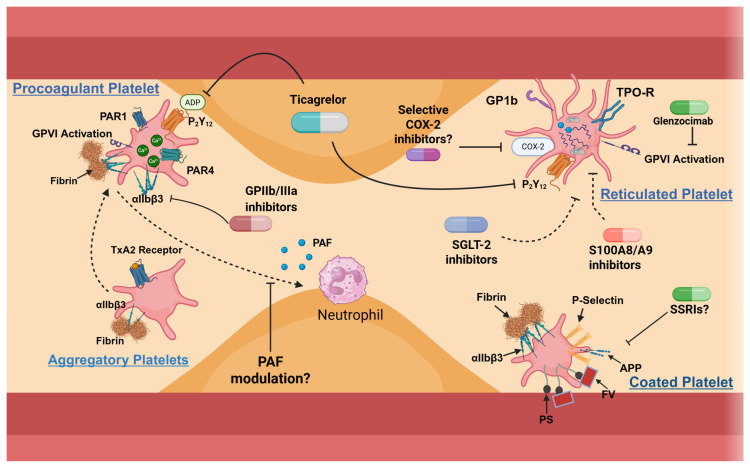
Schematic representation of distinct platelet subpopulations and their potential therapeutic modulation in coronary artery disease. The lesion is shown in yellow, and the endothelium in dark red. For coated platelets, modulation may be attempted with SSRIs, although bleeding risk remains a concern. Aggregatory platelets, under strong stimulation, can exhibit a procoagulant phenotype and release PAF, thereby stimulating neutrophils and contributing to atherosclerotic plaque development. Procoagulant and reticulated platelets could be targeted using reversible P_2_Y_12_ inhibitors such as ticagrelor, despite some associated bleeding concerns. GPIIb/IIIa inhibitors may be used for procoagulant platelets, while selective COX-2 inhibitors might be considered for reticulated platelets. SGLT2 inhibitors and S100A8/A9 are represented with dashed lines because they do not directly target reticulated platelets but may reduce the overall disease process and consequently the number of reticulated platelets (see text for more details). GPVI, glycoprotein VI; PAR1, protease-activated receptor 1; P_2_Y_12_, purinergic receptor P_2_Y_12_; PAR4, protease-activated receptor 4; GP ⍺IIbβ3, glycoprotein alpha IIb beta3; COX-2, cyclooxygenase 2; GP1b, glycoprotein 1b; TPO-R, thrombopoietin receptor; SGLT-2, sodium-glucose cotransporter-2; TxA_2_, thromboxane A_2_; PAF, platelet activating factor; PS, P-selectin; FV, factor V; APP, amyloid precursor protein; SSRIs, selective serotonin reuptake inhibitors.

The following section provides a summary of platelet subpopulations organized according to specific diseases and their corresponding potential targeted therapies (Figure 3). We have both summarized and expanded upon concepts discussed in previous sections, emphasizing the therapeutic perspective. This classification is primarily intended for didactic purposes, helping clinicians and medical staff gain a clearer understanding of the diverse platelet subpopulations and their relevance in disease management.

### 5.1. Targeting Reticulated Platelets

RPs have emerged as pivotal players in thromboinflammatory diseases and therapeutic resistance. Their rapid turnover undermines the efficacy of irreversible antiplatelet agents like aspirin, clopidogrel, and prasugrel (Table 2). In contrast, reversible P_2_Y_12_ inhibitors such as ticagrelor and cangrelor provide continuous receptor blockade and effectively inhibit newly formed RPs [121,122,123]. Recently it was demonstrated that reticulated platelets (RPs) in CAD patients exhibit enhanced GPVI and PI3K signaling, elevated activation markers, and unique non-coding RNA signatures. Functional studies confirm that RPs promote heightened aggregation and thrombus formation, which can be mitigated by PI3K or GPVI inhibition. These results identify targetable pathways through which RPs contribute to thrombosis and suggest that selective inhibition may reduce residual thrombotic risk, supporting the development of precision antiplatelet therapy in coronary artery disease [124]. In high-turnover states, residual COX-2 activity sustains TxA_2_ synthesis despite aspirin therapy, suggesting that selective COX-2 inhibition, if carefully timed, may enhance platelet suppression [60]. However, long-term use of COX-2 inhibitors could impair anti-inflammatory lipoxins [125]. Beyond thrombosis, RPs also contribute to cancer and metabolic disease. In non-small cell lung cancer, PD-L1-expressing RPs have been implicated in immune checkpoint resistance. This effect can be reversed by vascular endothelial growth factor (VEGF) or platelet-derived growth factor (PDGF) blockade or by the ⍺IIbβ3 inhibitor eptifibatide, which also restores T cell activity [126]. In diabetes, sodium-glucose cotransporter 2 (SGLT2) inhibitors like dapagliflozin reduce RP production and cardiovascular risk through insulin-independent mechanisms [127]. Additionally, the small-molecule S100A8/A9 inhibitor ABR-215757, originally developed for autoimmune disease, has shown promise in preclinical models by suppressing RP-driven atherogenesis [53,128].

### 5.2. Targeting Coated Platelets

Coated platelets, known for their strong procoagulant activity and surface retention of key proteins, have recently gained attention as potential therapeutic targets. Selective serotonin reuptake inhibitors (SSRIs) have been shown to significantly reduce coated-platelet production, likely by depleting dense granule serotonin, a critical mediator of their formation. This reduction correlates with the increased bleeding risk and decreased incidence of myocardial infarction observed in SSRI users, particularly among smokers. Although the precise molecular mechanisms remain to be fully elucidated, these observations suggest that modulation of coated-platelet potential may underlie part of the cardioprotective effects associated with SSRIs [132].

### 5.3. Targeting Procoagulant Platelets

Procoagulant platelets have a high thrombin-generating capacity, yet they remain untargeted by current antiplatelet therapies. While aspirin has no measurable effect, potent P_2_Y_12_ inhibitors like ticagrelor and prasugrel significantly suppress thrombin hypersensitivity and abnormal ADP responses, unlike clopidogrel, which may explain their superior clinical outcomes [110]. In cancer-associated thrombosis, particularly colorectal cancer, inhibiting PS exposure using agents such as lactadherin effectively reduces procoagulant activity and MP formation [112]. Additionally, selective blockade of key procoagulant regulators such as TMEM16F and cyclophilin D, or carbonic anhydrase inhibitors, may offer novel approaches to prevent thrombotic events like venous thromboembolism without compromising hemostasis [113]. In diabetes, a known prothrombotic state, αIIbβ3 inhibition reduces PS exposure and thrombin generation, though clinical trials with such inhibitors remain inconclusive [133,134]. AKT signaling pathway is associated with the formation of procoagulant platelets in severe COVID-19 patients without αIIbβ3 engagement. The inhibition of PI3K/AKT phosphorylation might represent a promising strategy to reduce the risk for thrombosis in patients with severe COVID-19 [120] (Table 3).

## 6. Conclusions

The growing recognition of platelet heterogeneity, including both young versus old platelets and functionally diverse subpopulations, has transformed our understanding of their roles in health and disease. For the latter group of functionally defined platelet subtypes, an important unresolved question is whether they originate solely through activation of circulating platelets or whether megakaryocytes release pre-committed subsets. Clarifying this will be essential to fully harness the clinical potential of these different platelet populations.

Different platelet subtypes carry out diverse and specialized roles, often contributing to disease mechanisms and limiting the effectiveness of conventional antiplatelet therapies. Their selective involvement in pathological processes highlights the opportunity to move beyond generalized approaches. Instead of treating all platelets as equal, emerging evidence supports more refined strategies that target harmful subpopulations while preserving normal hemostatic function. This shift toward precision targeting holds promise for improving patient outcomes and redefining the future of antiplatelet therapy.

## Figures and Tables

**Table 1:** The distinguishing features of aggregatory, coated (COAT), and procoagulant platelet subtypes. ADP, adenosine diphosphate; TG2, transglutaminase 2; PS, phosphatidylserine; TMEM16F, transmembrane protein 16 F; GPVI, glycoprotein VI; Fc receptor γ-chain receptor; mPTP, mitochondrial permeability transition pore; VWF, von Willebrand factor; FV, factor V.

Feature	Aggregatory	Coated (COAT)	Procoagulant
Biological roles	Form the thrombus core, ensuring cohesion and stability	Retain α-granule and plasma proteins linked to coagulation	Provide a platform for coagulation complex assembly and thrombin generation
Formation trigger	Classical platelet activation (e.g., ADP, thrombin)	Dual stimulation (e.g., thrombin + collagen)	Strong dual stimulation (thrombin + collagen) or regulated apoptosis/necrosis
Key receptors/proteins	Active αIIbβ3, P-selectin	Initially active αIIbβ3, GPVI/FcRγ, transglutaminases (FXIII, TG2), serotonin	PS exposure, αIIbβ3 inactivated (high-Ca^2+^ subset) or active (low-Ca^2+^ subset), TMEM16F, mPTP, Bak/Bax
Membrane integrity/morphology	Preserve membrane integrity, no PS exposure	Preserved integrity, PS exposure, surface protein retention	Loss of integrity (necrotic/apoptotic subsets), ballooning, PS exposure
Granules and content	Active α-granule secretion, fibrinogen/VWF adhesion	Retain FV, fibrinogen, VWF, thrombospondin, fibronectin, α2-antiplasmin	Retain procoagulant factors, release microparticles
Role under shear	Dominant under venous and moderate arterial shear; VWF important at high shear	Form under extreme vascular injury with collagen exposure + sustained thrombin generation	Active in thrombi across shear ranges; key in venous thrombi, trauma, cancer, diabetes

**Table 2:** Key pathophysiological mechanisms involving reticulated platelets, their associated biological pathways, and corresponding therapeutic interventions. RPs, reticulated platelets; P_2_Y_12_, purinergic receptor P_2_Y_12_; ACS, acute coronary syndromes; MI, myocardial infarction; GPVI, glycoprotein VI; PI3K, Phosphatidylinositol 3-kinase; COX-2, cyclooxygenase-2; TxA_2_, thromboxane A_2_; VEGF, vascular endothelial growth factor; PDGF, platelet-derived growth factor; NSCLC, non-small cell lung carcinoma; MPV, mean platelet volume; SGLT2, sodium-glucose cotransporter-2; IL, interleukin; IL-6, interleukin-6; TPO, thrombopoietin; HbA1C, hemoglobin A1C.

Mechanism/Problem	Underlying Biology	Therapeutic Strategy/Target	Rationale	Clinical Stage/Evidence Level
High platelet turnover → newly formed RPs	RPs are more reactive, preferentially recruited to thrombus cores.	Use reversible P_2_Y_12_ inhibitors (e.g., ticagrelor, cangrelor) instead of irreversible drugs (clopidogrel, prasugrel)	Switch to drugs with sustained plasma levels or reversible binding → Continuous inhibition of newly formed platelets; maintains antiplatelet effect despite turnover	PLATO trial (multicenter, randomized, double-blind): Ticagrelor vs. Clopidogrel in patients with ACS reduced death, MI, or stroke; no increase in major bleeding overall but increased non-procedure-related bleeding [129].
Hyperreactive reticulated platelets in CAD	Increased proteomic expression of GPVI, and PI3K-mediated signaling	GPVI inhibition (glenzocimab, revacept), PI3K inhibition (LY294002)	Targeting these pathways can mitigate RP hyperreactivity and thrombotic risk	Preclinical evidence from transcriptomic, proteomic, and functional studies [124] Phase II clinical trial in patients with stable ischemic heart disease undergoing elective PCI → revacept (anti-GPVI) was safe with few bleeding events but did not reduce myocardial injury [130].
COX-2–mediated thromboxane A_2_ production	RPs express higher COX-2, which is less sensitive to low-dose aspirin	COX-2 selective inhibition or adjusted aspirin dosing	Blocks TxA_2_ production in RPs, reducing residual platelet activation	While in vitro inhibition of platelet COX-2 reduced TxB2 [60], clinical data are mixed: in patients undergoing cardiac bypass surgery, COX-2 inhibition with celecoxib did not decrease thromboxane synthesis, highlighting the need for further investigation [58].
Reticulated platelets (RPs) contribute to cancer progression and immune evasion	In non-small cell lung cancer, RPs express PD-L1, which promotes immune checkpoint resistance	VEGF, PDGF, αIIbβ3 integrin (eptifibatide)	Blocking VEGF or PDGF, or inhibiting αIIbβ3, reverses RP-mediated immunosuppression and restores T cell activity	An exploratory study involving NSCLC patients (APOLLO study) demonstrated an initial association between anti-platelet and immune checkpoint inhibitor treatment and longer progression-free survival and a trend toward better overall survival [131]
Diabetes-associated reduced antiplatelet efficacy and enhanced thrombotic risk	Hyperglycemia → neutrophil S100A8/A9 → Kupffer cell IL-6 → hepatic TPO → megakaryocyte proliferation → RPs; elevated MPV and monocyte-platelet aggregates correlate with HbA1c	Glycemic control (SGLT2 inhibitors), S100A8/A9 inhibitors (ABR-215757), IL-6/TPO modulation	Reduces RPs production, platelet hyperactivity, and restores antiplatelet therapy effectiveness	The small-molecule S100A8/A9 inhibitor ABR-215757 has shown promise in preclinical models by suppressing RP-driven atherogenesis [53,128].

**Table 3:** The key mechanisms, biological features, and therapeutic strategies targeting procoagulant platelets across a range of disease states. HIT, heparin-induced thrombocytopenia; FcγRIIa, Fc gamma receptor IIa; PS, phosphatidylserine; MPs, microparticles; FXa, activated factor X; DVT, deep vein thrombosis; PE, pulmonary embolism; TMEM16F, transmembrane protein 16F; FVa, factor Va; PI3K, phosphoinositide 3-kinase; AKT, protein kinase B; CD62P, P-selectin; VITT, vaccine-induced immune thrombotic thrombocytopenia; PF4, platelet factor 4.

Mechanism/Problem	Underlying Biology	Therapeutic Strategy/ Target	Rationale	Clinical Stage/Evidence Level
HIT	HIT antibodies activate platelets via FcγRIIa in a heparin-dependent manner, enhancing thrombin generation	Target FcγRII⍺	Blocking FcγRIIa or inhibiting procoagulant platelets can reduce thrombotic risk	VIB9600 (humanized anti-FcγRIIA): shown effective and safe in preclinical primate studies; currently investigational for immune-mediated inflammatory conditions (e.g., sepsis) [135].
Hypercoagulable state in colon cancer	PS-exposing platelets and MPs increase with cancer stage, promoting FXa and thrombin generation	PS blockade (lactadherin)	Inhibiting PS exposure reduces clotting and hypercoagulability	In advanced (stage II–IV) colon cancer, antiplatelet therapy insufficient; PS inhibition remains investigational in preclinical settings [112].
Venous thrombosis (DVT/PE)	Procoagulant platelets enriched in thrombi; regulators TMEM16F and cyclophilin D control platelet procoagulant activity	Pharmacological inhibition (methazolamide), genetic targeting of TMEM16F/cyclophilin D	Selective inhibition reduces thrombus formation while preserving normal hemostasis	MZA effective in vitro and in vivo (reduced PS exposure and arterial thrombosis in preclinical studies); potential hematocrit-related side effects need further evaluation [113].
Trauma-induced platelet dysfunction	Histone H4 triggers platelet ballooning, PS exposure, and microparticle release, promoting thromboinflammation	Target histone-mediated platelet activation or procoagulant signaling	Reducing procoagulant platelet formation may limit coagulopathy and inflammation after trauma	Targeting histone-mediated platelet activation or procoagulant signaling remains hypothesis-driven and will need further investigations [114].
Diabetes-associated hypercoagulability	Platelets show increased PS exposure, factor Va binding, microparticle release; small platelets exhibit spontaneous procoagulant activation	Glycoprotein ⍺IIbβ3 blockade	Reduces PS exposure, FVa binding, and thrombin generation, lowering thrombotic risk in diabetics	Chronic GPIIb/IIIa inhibition in T2DM remains preclinical/exploratory [116], with previous oral inhibitors failing in ACS due to safety concerns [133,134].
COVID-19 thromboinflammatory complications	Dysregulated procoagulant platelet responses; PI3K-AKT signaling mediates PS and CD62P expression; cyclophilin D essential for procoagulant formation	PI3K/AKT inhibitors, targeting cyclophilin D or FcγRIIa	Modulation of procoagulant platelet pathways may reduce microvascular thrombosis and thromboinflammatory damage	BAY1125976 and BYL719 (PI3K/AKT inhibitors) have been shown to be effective in preclinical/experimental settings [120].
VITT	Anti-PF4 antibodies trigger platelet procoagulant activation, PS exposure, and P-selectin upregulation	Target PF4-antibody interaction, FcγRIIa, or procoagulant platelet formation	Prevents formation of procoagulant platelets and widespread thrombosis	Preclinical investigation [119].

## Data Availability

No new data were created or analyzed in this study.
